# Synthesis, Evaluation of Antioxidant Activity and Crystal Structure of 2,4-Dimethylbenzoylhydrazones

**DOI:** 10.3390/molecules180910912

**Published:** 2013-09-05

**Authors:** Muhammad Taha, Nor Hadiani Ismail, Waqas Jamil, Sammer Yousuf, Faridahanim Mohd Jaafar, Muhammad Imran Ali, Syed Muhammad Kashif, Ejaz Hussain

**Affiliations:** 1 Atta-ur-Rahman Institute for Natural Product Discovery, Universiti Teknologi MARA, Puncak Alam Campus, Bandar Puncak Alam 42300, Selangor, Malaysia; E-Mail: mia318.iccs@yahoo.com; 2 Faculty of Applied Science Universiti Tecknologi MARA, Shah Alam 40450, Selangor, Malaysia; E-Mails: norhadiani@salam.uitm.edu.my (N.H.I.); mfaridahanim@yahoo.com (F.M.J.); 3 Institute of Advance Research Studies in Chemical Sciences, University of Sindh Jamshoro, Hyderabad 76080, Pakistan; E-Mails: waqas143kh@yahoo.com (W.J.); hope_cancer@yahoo.com (S.M.K.); 4 H.E.J. Research Institute of Chemistry, International Center for Chemical and Biological Sciences, University of Karachi, Karachi 75270, Pakistan; E-Mails: dr.sammer.yousuf@gmail.com (S.Y.); ejaz.iccbs@gmail.com (E.H.)

**Keywords:** 2,4-dimethylbenzoylhydrazones, DPPH scavenging, superoxide ion scavenger, crystal structure

## Abstract

2,4-Dimethylbenzoylhydrazones **1**–**30** were synthesized by condensation reactions of 2,4-dimethylbenzoylhydrazide with various aromatic aldehydes and characterized. The assigned structures of compounds **10**, **15** and **22** were further supported by single-crystal X-ray diffraction data. The synthesized compounds were evaluated for their *in vitro* DPPH radical scavenging activity. They exerted varying degree of scavenging activity toward DPPH radical with IC_50_ values between 25.6–190 µM. Compounds **1**, **4**, **2**, **3**, **7**, and **6** have IC_50_ values of 25.6, 28.1, 29.3, 29.8, 30.0 and 30.1 µM respectively, showing better activity than an *n*-propyl gallate standard (IC_50_ value = 30.30 µM). For super oxide anion scavenging activity compounds **1**, **2** and **3** with IC_50_ values of 98.3, 102.6, and 105.6, respectively, also showed better activity than the *n*-propyl gallate standard (IC_50_ value = 106.34 µM).

## 1. Introduction

Hydrazone Schiff bases are a versatile set of compounds having unique properties. Hydrazones are the bimolecular condensation product of an aryl or alkyl hydrazine and a carbonyl compound (aldehyde or ketone). Due to their synthetic simplicity and active pharmacophore group, *i.e.*, the C=N moiety, hydrazones are found to display biological and catalytic activities. One of the most prevalent *in vivo* biochemical processes involving the formation of a Schiff base is the condensation of lysine residues with the carbonyl group of methylglyoxal [1]. In this respect the Schiff base derived from pyrodoxal and amino acids has been thoroughly studied [2]. A broad range of biological activities of hydrazones such as anticancer [3] antibacterial [4] antifungal, herbicidal [5,6], anti-convalescent [7], anti-oxidant [8], and diuretic properties [9,10] have been reported. Several hetrocyclic hydrazones have been found to be potent cytotoxic [11], antimalarial [12], antiproliferative [13], anticancer and antifungal agents [14]. Hydrazones are also well known for their applications in foods and dyes as well as the agrochemical industry [15,16]. Among the various hydrazones, hydrazide-hydrazones merit special attention due to their distinct structural features. They have been reported as antioxidant [17,18,19] antifungal [20], anti-depressant [21], vasodilating [22], and anthelmintic agents [23]. Nifuroxazide, a hydrazide hydrazone has been patented as an intestinal antiseptic. Some of the more effective anti-tuberculosis drugs like iproniazide and isocarboxazide also contain hydrazide-hydrazone moieties [24]. A hydrazide hydazone derived from safrole was found to be s potent antiinflammatory/antinociceptive agent [25], while benzylidene 10*H*-phenothiazine-1-carbohydrazide has also been reported as an important anti-platelet agent [26]. The active pharmacophore (-CONH-N=CH-) of hydrazide hydrazones is mainly responsible for the significant biological activities although the attached neighboring groups may also be responsible [27]. Metal complexes of hydrazide hydrazones are also biologically active, besides having an important role in catalytic chemistry [28,29,30,31]. These metal complexes showed remarkable anti-cancer, antibacterial and antifungal activities [32].

Free radicals are highly reactive compounds formed in the body during biochemical reactions. They are oxidized the other metabolites in the body and can causes diseases such as Alzheimer’s disease (AD). The main reason for AD is oxidative stress, so it is necessary to inhibit or stop the formation of free radicals. Anti-oxidant compounds either natural of synthetic can be used to prevent the formation of free radicals. The major role of anti-oxidant compounds is to inhibit or neutralize the free radicals as well as repair the damages caused by free radicals [33].

In the view of the above, a series of structurally similar 2,4-dimethylbenzoylhydrazones were synthesized and evaluated for their antioxidant potential. This study has revealed some potential leads for possible pharmaceutical applications and further investigation may help in the development of new anti-oxidative agents for important metabolic functions.

## 2. Results and Discussion

### Chemistry

**Scheme 1:**
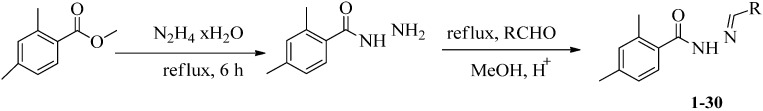
Synthesis of 2,4-dimethylbenzoylhydrazones.

In the continuation of our work on benzoylhydrazide [34,35,36], 2,4-dimethylbenzoylhydrazones **1**–**30** were synthesized from 2,4-dimethylbenzoylhydrazide, which in turn was synthesized from methyl 2,4-dimethylbenzoate by refluxing with hydrazine hydrate for 4 h. The 2,4-dimethylbenzoylhydrazide obtained was recrystallized from methanol to afford good yields of product. Next, 2,4-dimethylbenzoylhydrazones were prepared through the condensation reactions of 2,4-dimethylbenzoylhydrazide with different aromatic aldehydes in the presence of acetic acid (Scheme 1). The reaction mixture was refluxed in ethanol for 3 to 4 h. The crude products were recrystallized from methanol to give needle-like crystal in most cases (all yields are reported as crude product). Compounds **1**–**3**, **7**, **12**, **14**–**16**, **20**, and **29** are new, while compounds **4**–**6**, **8**–**11**, **13**, **17**, **18**, **21**, **23**–**28**, and **30** showed only CAS registry number with zero reference. Only compound **19** and **22** reported in the literature.

The structural confirmation of 2,4-dimethylbenzoylhydrazones **1**–**30** was accomplished by several spectroscopic techniques, including ^1^H-NMR and mass spectroscopic. All compounds have an *E*-configuration as recently reported for some similar products [37,38,39,40,41,42]. We are also reporting three new crystal structures of compounds **10**, **15** and **22**.

## 3. Antioxidant Activity

### 3.1. DPPH Scavenging Activity

2,4-Dimethylbenzoylhydrazones **1**–**30** were evaluated for their *in vitro* DPPH radical scavenging activity and showed varying degree of activity, with IC_50_ values between 25.6–190 µM. Compounds **1**, **4**, **2**, **3**, **7**, and **6** have IC_50_ values of 25.6, 28.1, 29.3, 29.8, 30.0 and 30.1 µM, respectively, showing better activity than standard *n*-propyl gallate IC_50_ value = 30.30 µM. Compound **5** (IC_50_ = 34.1 µM), **8** (IC_50_ = 34.3 µM), **12**, (IC_50_ = 34.2 µM), and **9** (IC_50_ = 40.1 µM) exhibited good activity comparable to the standard. On the other hand, compounds **14** and **11**, with IC_50_ values of 52.2 and 60.1 µM, respectively, showed only moderate activities. Compounds **20**, **29** and **27** were found to be weakly active. Compounds **10**, **13**, 1**5**–**19**, **21**–**26**, **28** and **30**, showed less than 50% activity; and thus their IC_50_ values were not evaluated (Table 1).

By comparing structural information and antioxidant activity, it was observed that DPPH radical scavenging antioxidant activity depends on two parameters: the functional groups on aromatic ring, and the position of functional groups on the ring. It was observed that the active compounds of the series bear an -OH group on the ring and activity pattern is such that, the more the -OH groups, the activity will be higher. Thus, the tri-OH group-containing derivatives were found to be more active than di- and mono-OH substituted analogues. Compound **1**, which is a 3,4,5-trihydroxy derivative was the most active compound of this series, while compound **2** which bears a 2,4,6-trihyroxy group showed only slightly low activity than compound **1**. Two factors might thus be involved in this activity pattern, one is the position of -OH groups and second, the extra resonance stability of compound **1** compared to compound **2** [43].

**Table 1:** *In vitro* DDPH Radical Scavenging Activity of Compounds **1**–**30**.

Comp.	R	Yield	IC_50_	Comp. No.	R	Yield	IC_50_
No.		(%)	(µM ± SEM ^a^)			(%)	(µM ± SEM ^a^)
**1**	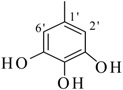	84	25.6 ± 0.5	**10**	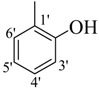	88	NA ^b^
**2**	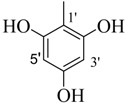	82	29.3 ± 1.0	**11**	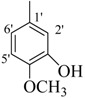	90	60.1 ± 2.2
**3**	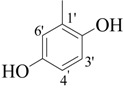	78	29.8 ± 1.1	**12**	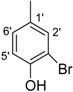	87	34.2 ± 2.1
**4**	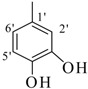	84	28.14 ± 0.8	**13**	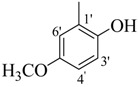	90	NA ^b^
**5**	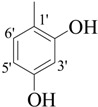	85	34.1 ± 1.0	**14**	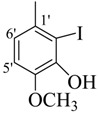	87	52.2 ± 2.8
**6**	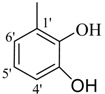	86	30.1 ± 1.3	**15**	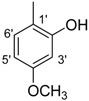	82	NA ^b^
**7**	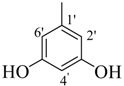	81	30.0 ± 1.2	**16**	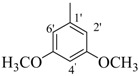	82	NA ^b^
**8**	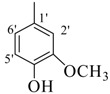	83	34.3 ± 1.5	**17**	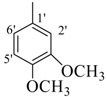	84	NA ^b^
**9**	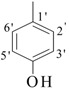	92	40.0 ± 1.8	**18**	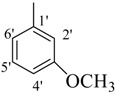	83	NA ^b^
**19**	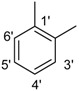	85	NA ^b^	**25**	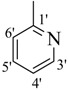	82	NA ^b^
**20**	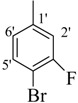	87	130.0 ± 4.8	**26**	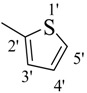	88	NA ^b^
**21**	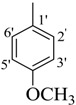	88	NA ^b^	**27**	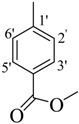	90	190.0 ± 5.1
**22**	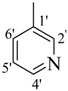	90	NA ^b^	**28**	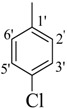	92	NA ^b^
**23**	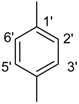	92	NA^b^	**29**	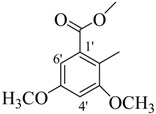	91	180.0 ± 4.6
**24**	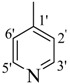	90	NA^b^	**30**	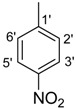	84	NA^b^
**Standard drug *n*-propyl gallate ^c^**				30.30 ± 0.2			

^a^ SEM is the standard error of the mean; ^b^ NA = Not active; ^c^ *n*-propyl gallate standard used for the DDPH radical scavenging activity assays.

Interesting results are obtained when we compare the dihydroxy derivatives. Five dihydroxy derivatives showed excellent activity and variation in their activity is mainly due to the stability of the resulting radical. Compounds **4**, **3**, **6**, and **7** showed excellent activity, better than standard. Interestingly, compound **5** having a 2,4-dihydroxy group, is the only dihydroxy analogue which has an IC_50_ value above the standard, indicating less stability of its radical as compared to other analogues [44,45].

Their activities of monohydroxy compounds **8**–**15** mainly depends upon the position of the hydroxy group. Compounds **8**, **9** and **12** showed good activity having a 4-hydroxy group and the variation in their activities is due to the presence of a 3-methoxy as in compound **8** and a bromine in compound **12**, which showed almost the same activity, and may be due to the resonance stabilizing potential of the methoxy and bromine substituents. On the other hand compounds **11** and **14** have a 3-hydroxy along with other sustituents but still showed less activity than the *para*-substituted analogues. It is well established that *para*-substitution has more stabilizing potential then *meta-*substitution. Compounds **10**, **13** and **15** having 2-hydroxy group are found to be inactive, which may be due to the intramolecular hydrogen bonding. Compounds **20**, **27** and **29** have halogen and ester moieties, respectively, which weakly stabilized the radical and showed weak activity. Compounds **10**, **13**, 1**5**–**19**, **21**–**26**, **28** and **30**, not having any substituent to help stabilize the radical effectively, were found to be inactive as expected.

### 3.2. Superoxide Scavenging Activity

Compounds **1**, **2** and **3** showed IC_50_ values of 98.3, 102.6 and 105.6 μM, respectively, better than the *n*-propyl gallate standard (106.3 μM). Compounds **4**, **5**, **6**, **7**, **8**, **9**, **12** and **14** showed moderate activity respectively (145, 170.2, 175.0, 180.1, 190.1, 208.9, 210.1 and 260.3 μM). Compound **20** showed very weak activity (Table 2).

**Table 2:** *In vitro* Superoxide Anion Radical Scavenging Activity of Compounds **1**–**30**.

Comp. No.	IC_50_ (μM ± SEM ^a^)	Comp. No.	IC_50_ (μM ± SEM ^a^)
**1**	98.3 ± 1.2	**16**	NA ^b^
**2**	102.6 ± 1.5	**17**	NA ^b^
**3**	105.6 ± 1.7	**18**	NA ^b^
**4**	145 ± 2.1	**19**	NA ^b^
**5**	170.2 ± 3.2	**20**	315.1 ± 8.4
**6**	175.0 ± 3.5	**21**	NA ^b^
**7**	180.1 ± 3.8	**22**	NA ^b^
**8**	190.1 ± 3.9	**23**	NA ^b^
**9**	208.9 ± 5.4	**24**	NA ^b^
**10**	NA ^b^	**25**	NA ^b^
**11**	NA ^b^	**26**	NA ^b^
**12**	210.1 ± 4.4	**27**	NA ^b^
**13**	NA ^b^	**28**	NA ^b^
**14**	260.3 ± 6.4	**29**	NA ^b^
**15**	NA ^b^	**30**	NA ^b^
*n*-propyl gallate ^c^		106.34 ± 1.6	

^a^ SEM is the standard error of the mean, ^b^ NA = Not active, ^c^ *n*-propyl gallate was the standard drug for the superoxide anion radical scavenging assays.

Compounds **8**, **10**, **11**, **13**, **15**–**19** and **21**–**30** showed less than 50% inhibition and therefore the IC_50_ values were not further evaluated. Compounds **1** and **2** have trihydroxy substitution pattern at ring A, and showed remarkable activity, more active than the standard. Among the five dihydroxy-substituted analogues only compound **3** showed better activity than the standard, while the rest of the dihydroxy derivatives were found to be only moderately active towards superoxide. Compound **9** having a 4-hydroxy at ring A, showed moderate activity. Surprisingly, compound **8** having a 3-methoxy-4-OH substituted showed better activity than compound **9**, while replacement of -OCH_3_ with -Br, decreased the activity. The weak activity of compound **14** might be due to combined effect of iodine and hydroxyl groups. The dihalogenated compound **20** also showed weak activity.

## 4. Experimental

### 4.1. General

NMR experiments were performed on an Ultra Shield Bruker FT NMR 500 MHz instrument (Wissembourg Cedex, France). CHN analysis was performed on a Carlo Erba Strumentazion-Mod-1106 (Milan, Italy). Electron impact mass spectra (EI MS) were recorded on a Finnigan MAT-311A unit (Bremen, Germany). Thin layer chromatography (TLC) was performed on pre-coated silica gel aluminum plates (Kieselgel 60, 254, E. Merck, Darmstadt, Germany). Chromatograms were visualized by UV at 254 and 365 nm.

### 4.2. X-ray Crystallography Studies

**Figure 1:**
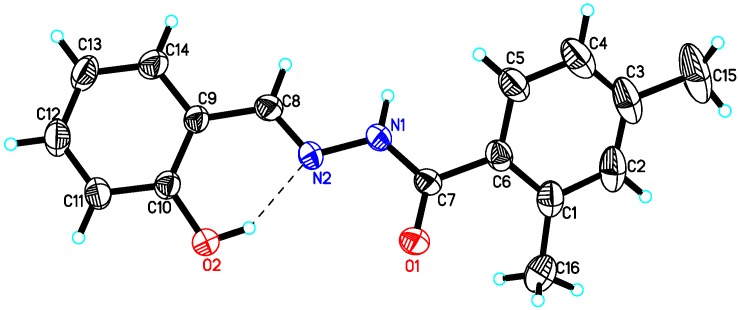
ORTEP view of compound **10** with displacement ellipsoids drawn at 30% probability level. Dashed lines represent the intermolecular interaction; selected bond lengths [Å]: C7-O1 1.229(2), O2-C10 1.351(2), N1-C7 1.348(2), N1-N2 1.382(19), N2-C8 1.276(2).

**Figure 2:**
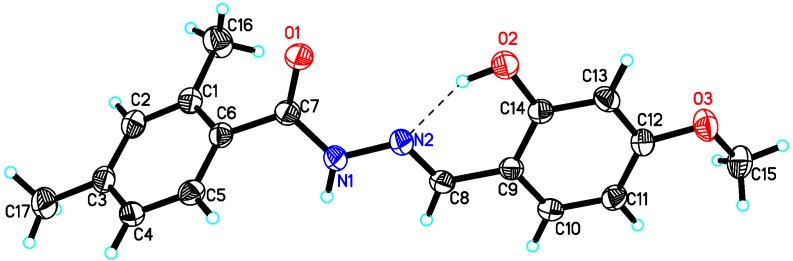
ORTEP view of compound **15** with displacement ellipsoids drawn at 30% probability level. Dashed lines represent the intermolecular interaction; selected bond lengths [Å]: C7-O2 1.226(18), O2-C14 1.349(2), O3-C12 1.357(2), N1-C7 1.352(2), N1-N2 1.38(19), N2-C8 1.276(2).

**Figure 3:**
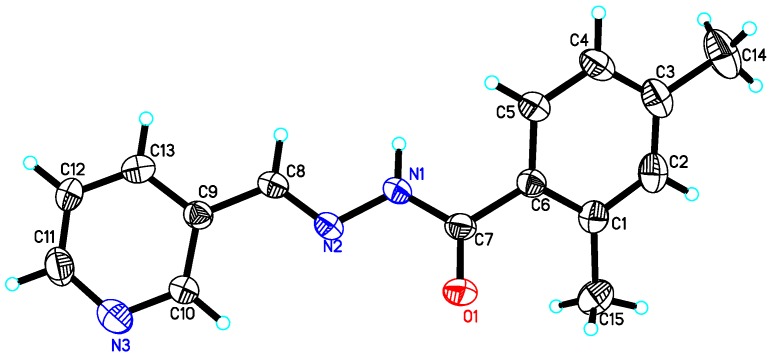
ORTEP view of compound **22** with displacement ellipsoids drawn at 30% probability level; selected bond lengths [Å]: C7-O1 1.228(2), N1-C7 1.351(3), N1-N2 1.380(2), N2-C8 1.269(3).

The structures of compounds **10**, **15** and **22** were further supported by single crystal X-ray diffraction analysis. The **10**, **15** and **22** were found to be monoclinic system crystals with space groups P21/c (compounds **10** and **22**) and P21/n (compound **15**). The ORTEP views of compounds **10** (Figure 1), **15** (Figure 2) and **22** (Figure 3), clearly indicated that structures of all compounds were composed of two planar phenyl rings (C1—C6 and C9—C14) with an *E-*configuration azomethine double bond. The hydroxyl functionality of the phenyl ring played an important role in stabilizing the *E-*configuration of the azomethine olefin bond in compounds **10** and **15** via intramolecular interaction. The crystal details of compounds **10**, **15** and **22** as well as experimental data are summarized in Table 3. Single crystal X-ray diffraction data was collected on Bruker Smart APEX II, CCD area detector diffractometer [46] followed by data reduction preformed by SAINT program. The structure was solved and expanded by direct method and Fourier transformation techniques, respectively. SHELXL97 program was used to refine the structure by using full-matrix least-square calculation on *F^2^* [47] (Sheldrick 1997). The ORTEP program was used to plot the Figure 1, Figure 2 and Figure 3. Figures were plotted with the aid of ORTEP program [48]. Crystallographic data of compound **10** (CCDC 933686), **15** (CCDC 933687) and **22** (CCDC 933685) can be obtained from Cambridge Crystallographic Data Center without any cost.

**Table 3:** The crystal and experimental data of compounds **10**, **15** and **22**.

	Compound 10	Compound 15	Compound 22
Empirical formula	C_16_H_16_N_2_O_2_	C_17_H_18_N_2_O_3_	C_15_H_15_N_1_O_3_
Formula weight	268.31	298.33	253.30
Temperature	273(2)	273(2)	273(2)
Wavelength	0.71073 Å	0.71073 Å	0.71073 Å
Crystal system	Monoclinic	Monoclinic	Monoclinic
Space group	P21/c	P21/n	P21/c
A	12.7293(17) Å	11.1984(9) Å	14.0960(14) Å
B	13.0700(17) Å	10.0703(9) Å	11.8440(12) A
C	8.8762(12) Å	13.8665(12) Å	8.2813(8) Å
*β*	94.845(3)°	102.535(2)°	100.889(2)°
Volume	1,463.2(3) A^3^	1,526.5(2) A^3^	1,357.7(2) A^3^
Z	4	4	4
Calculated density	1.218 mg/m^3^	1.298 mg/m^3^	1.239 mg/m^3^
Absorption coefficient	0.082 mm^−1^	0.090 mm^−1^	0.080 mm^−1^
F(000)	568	632	536
Crystal size	0.52 × 0.12 × 0.11 mm	0.56 × 0.43 × 0.12 mm	0.56 × 0.18 × 0.13 mm
θ range	2.24 to 25.49°	2.13 to 25.49°	1.47 to 25.40°
Reflections Collected	8546	8849	7827
Reflections Unique	2713	2758	2522
(*R*_int_)	0.0275	0.0212	0.0313
*R*_1_ with I > 2σ(I)	0.0463	0.0438	0.0536
*R*_2_ with I > 2σ(I)	0.1124	0.1248	0.1508
*R*_1_ for all data	0.0774	0.0520	0.0702
*R*_2_ for all data	0.1323	0.1337	0.1607
Goodness of fit	1.030	1.057	1.014
max/min *ρ* eA˚^−3^	0.140 and −0.110	0.232 and −0.018	0.265 and −0.258

### 4.3. DPPH (1,1-Diphenyl-2-picryl hydrazyl) Free Radical Scavenging Activity

The free radical scavenging activity was measured by 1,1-diphenyl-2-picrylhydrazyl (DPPH) using literature protocols. The reaction mixture contained test sample (5 μL, 1 mM in DMSO) and DPPH (Sigma, 95 μL, 300 μM) in ethanol. The reaction mixture was taken into a 96-well microtiter plate and incubated at 37 °C for 30 min. The absorbance was measured at 515 nm using microtiter plate reader (Molecular Devices, Sunnyvale, CA, USA). Percent radical scavenging activity was determined in comparison with DMSO containing control (Table 1). IC_50_ values represent the concentration of compounds able to scavenge 50% of DPPH radicals. Propyl gallate was used as positive control. All chemicals used were of analytical grade (Sigma, Ronkonkoma, NY, USA).

### 4.4. *In Vitro* Assay for Superoxide Anion Radical Scavenging Activity

The superoxide producing system was set up by mixing phenazinemethosulfate (PMS), NADH, and oxygen (air), and the production of superoxide was estimated by the nitroblue tetrazolium method. Measurement of superoxide radical scavenging activity was carried out on the basis of the method described by the modified method used by Ferda. In aerobic reaction mixtures containing NADH, phenazine methosulphate and nitro blue tetrazolium, PMS is reduced by NADH and then gave rise to O_2_^−^, which in turn reduced NBT. On the basis of this PMS has frequently been used to mediate O_2_^−^.

The reaction mixture comprised 100 µΜ β-nicotinamide adenine dinucleotide reduced form (NADH, 40 µL), 80 µM of nitro blue tetrazolium (NBT, 40 µL), 8 µM phenazine methosulphate (PMS, 20 µL), 1 mM sample (10 µL), and 0.1 M phosphate buffer (pH 7.4, 90 µL). The reagents were prepared in buffer and sample in DMSO. The reaction was performed in 96-well microtitre plate at room temperature and absorbance was measured at 560 nm. The formation of superoxide was monitored by measuring the formation of water soluble blue formosan dye. A lower absorbance of reaction mixture indicated a higher scavenging activity of the sample. Percent radical scavenging activity (% RSA) by samples was determined in comparison with a control using the following equation:

%RSA = 100 − {(OD test compound/OD control) × 100

### 4.5. General Procedure for the Synthesis of 2,4-Dimethylbenzohydrazide

Methyl 2,4-dimethylbenzoate (40 mmol) was refluxed with hydrazine hydrate (10 mL) in methanol (50 mL) for 6 h. Excess hydrazine and methanol was evaporated to obtain crude product which was recrystallized from methanol to give pure 2,4-dimethylbenzohydrazide (92% yield).

### 4.6. General Procedure for the Synthesis of 2,4-Dimethylbenzohydrazones

The 2,4-dimethylbenzohydrazones were synthesized by refluxing in methanol (15mL) for 3 h each pure 2,4-dimethylbenzohydrazide (2 mmol) and various aryl aldehydes (2 mmol) in the presence of a catalytical amount of acetic acid. The progress of reaction was monitored by TLC. After completion of reaction, the solvent was evaporated by vacuum to afford crude products which were further recrystallized from methanol and got needle like pure product in good to excellent yields.

*(E)-N'-(3,4,5-Trihydroxybenzylidene)-2,4-dimethylbenzohydrazide* (**1**). Yield: 0.504 g (84%); ^1^H-NMR (DMSO-*d_6_*): δ 11.34 (s, 1H, NH), 9.07 (br. s, 3H, 3× OH), 8.02 (s, 1H, ArCH=N-R), 7.33 (d, 1H, *J*_6,5_ = 7.5 Hz, H-6), 7.11 (s, 1H, H-3), 7.01 (d, 1H, *J*_5,6_ = 7.5 Hz, H-5), 6.66 (s, 2H, H-2', H-6'), 2.37 (s, 3H, CH_3_), 2.32 (s, 3H, CH_3_); IR (KBr, cm^−1^): 3460 (OH), 3320 (N-H), 1652 (C=O), 1628 (C=N), 1248 (C-N); Anal. Calcd for C_16_H_16_N_2_O_4_, C = 63.99, H = 5.37, N = 9.33, O = 21.31, Found C = 64.01, H =5.37, N = 9.34, O = 21.32; EI MS *m/z* (% rel. abund.): 300.

*(E)-N'-(2,4,6-Trihydroxybenzylidene)-2,4-dimethylbenzohydrazide* (**2**). Yield: 0.492 g (82%); ^1^H-NMR (DMSO-*d_6_*): δ 11.10 (s, 1H, NH), 9.46 (br. s, 3H, 3× OH), 8.30 (s, 1H, ArCH=N-R), 7.28 (d, 1H, *J*_6,5_ = 8.0 Hz, H-6), 7.09 (s, 1H, H-3), 6.98 (d, 1H, *J*_5,6_ = 8.0 Hz, H-5), 6.78 (s, 2H, H-3', H-5'), 2.36 (s, 3H, CH_3_), 2.30 (s, 3H, CH_3_); IR (KBr, cm^−1^): 3480 (OH), 3370 (N-H), 1658 (C=O), 1632 (C=N), 1253 (C-N); Anal. Calcd for C_16_H_16_N_2_O_4_, C = 63.99, H = 5.37, N = 9.33, O = 21.31, Found C = 64.02, H = 5.35, N = 9.32, O = 21.33; EI MS *m/z* (% rel. abund.): 300.

*(E)-N'-(2,5-Dihydroxybenzylidene)-2,4-dimethylbenzohydrazide* (**3**). Yield: 0.443 g (78%); ^1^H-NMR (DMSO-*d_6_*): δ 11.78 (s, 1H, NH), 10.40 (s, 1H, OH), 8.96 (s, 1H, OH), 8.41 (s, 1H, ArCH=N-R), 7.39 (d, 1H, *J*_6,5_ = 8.5 Hz, H-6), 7.14 (s, 1H, H-3), 7.10 (d, 1H, *J*_5,6_ = 8.5 Hz, H-5), 6.93 (s, 1H, H-6'), 6.74–6.70 (m, 2H, H-3', H-4'), 2.37 (s, 3H, CH_3_), 2.32 (s, 3H, CH_3_); IR (KBr, cm^−1^): 3410 (OH), 3330 (N-H), 1656 (C=O), 1636 (C=N), 1256 (C-N); Anal. Calcd for C_16_H_16_N_2_O_3_, C = 67.59, H = 5.37, N = 9.85, O = 16.88, Found C = 67.58, H = 5.68, N = 9.84, O = 16.89; EI MS *m/z* (% rel. abund.): 284.

*(E)-N'-(3,4-Dihydroxybenzylidene)-2,4-dimethylbenzohydrazide* (**4**) [37]. Yield: 0.477 g (84%); ^1^H-NMR (DMSO-*d_6_*): δ 11.20 (s, 1H, NH), 9.10 (s, 2H, 2× OH), 8.10 (s, 1H, ArCH=N-R), 7.33 (d, 1H, *J*_6,5_ = 7.5 Hz, H-6), 7.21 (s, 1H, H-2') 7.11 (s, 1H, H-3), 7.09 (d, 1H, *J*_5,6_ = 7.5 Hz, H-5), 6.09 (d, 1H, *J*_5',6'_ = 8.5 Hz, H-5'), 6.78 (d, 1H, *J*_6',5'_ = 8.5 Hz, H-6'), 2.35 (s, 3H, CH_3_), 2.32 (s, 3H, CH_3_); IR (KBr, cm^−1^): 3405 (OH), 3330 (N-H), 1658 (C=O), 1634 (C=N), 1250 (C-N); Anal. Calcd for C_16_H_16_N_2_O_3_, C = 67.59, H = 5.37, N = 9.85, O = 16.88, Found C = 67.58, H = 5.68, N = 9.84, O = 16.89; EI MS *m/z* (% rel. abund.): 284.

*(E)-N'-(2,4-Dihydroxybenzylidene)-2,4-dimethylbenzohydrazide* (**5**). Yield: 0.482 g (85%); ^1^H-NMR (DMSO-*d_6_*): δ 11.7 (s, 1H, NH), 11.45 (s, 1H, OH), 9.91 (s, 1H, OH), 8.36 (s, 1H, ArCH=N-R), 7.38 (d, 1H, *J*_6,5_ = 7.5 Hz, H-6), 7.27 (d, 1H, *J*_6',5'_ = 6.5 Hz, H-6'), 7.11 (s, 1H, H-3), 7.08 (d, 1H, *J*_5,6_ = 7.5 Hz, H-5), 6.36 (dd, 1H, *J*_5',3'_ = 2.0, *J*_5',6'_ = 6.5 Hz, H-5'), 6.32 (d, 1H, *J*_3',5'_ = 2.0 Hz, H-3'), 2.37 (s, 3H, CH_3_), 2.33 (s, 3H, CH_3_); IR (KBr, cm^−1^): 3445 (OH), 3325 (N-H), 1657 (C=O), 1631 (C=N), 1251 (C-N); Anal. Calcd for C_16_H_16_N_2_O_3_, C = 67.59, H = 5.37, N = 9.85, O = 16.88, Found C = 67.58, H = 5.68, N = 9.84, O = 16.89; EI MS *m/z* (% rel. abund.): 284.

*(E)-N'-(2,3-Dihydroxybenzylidene)-2,4-dimethylbenzohydrazide* (**6**). Yield: 0.488 g (86%); ^1^H-NMR (DMSO-*d_6_*): δ 11.2 (s, 1H, NH), 9.60 (s, 2H, 2× OH), 8.45 (s, 1H, ArCH=N-R), 7.41 (d, 1H, *J*_6,5_ = 7.5 Hz, H-6), 7.14 (s, 1H, H-3), 7.10 (d, 1H, *J*_5,6_ = 7.5 Hz, H-5), 6.94 (d 1H, *J*_6',5'_ = 6.5 Hz, H-6'), 6.86 (d, 1H, *J*_4',5'_ = 7.0 Hz, H-4'), 6.36 (dd, 1H, *J*_5',4'_ = 7.0, *J*_5',6'_ = 6.5 Hz, H-5') 2.38 (s, 3H, CH_3_), 2.33 (s, 3H, CH_3_); IR (KBr, cm^−1^): 3420 (OH), 3335 (N-H), 1657 (C=O), 1632 (C=N), 1254 (C-N); Anal. Calcd for C_16_H_16_N_2_O_3_, C = 67.59, H = 5.37, N = 9.85, O = 16.88, Found C = 67.58, H = 5.68, N = 9.84, O = 16.89; EI MS *m/z* (% rel. abund.): 284.

*(E)-N'-(3,5-Dihydroxybenzylidene)-2,4-dimethylbenzohydrazide* (**7**). Yield: 0.460 g (81%); ^1^H-NMR (DMSO-*d_6_*): δ 11.30 (s, 1H, NH), 10.50 (s, H, OH), 9.52 (s, H, OH), 8.40 (s, 1H, ArCH=N-R), 7.40 (d, 1H, *J*_6,5_ = 7.5 Hz, H-6), 7.18 (s, 1H, H-3), 7.12 (d, 1H, *J*_5,6_ = 7.5 Hz, H-5), 6.95 (s, 2H, H-2', H-6'), 6.72 (s, 1H, H-4'), 2.38 (s, 3H, CH_3_), 2.34 (s, 3H, CH_3_); IR (KBr, cm^−1^): 3435 (OH), 3330 (N-H), 1649 (C=O), 1627 (C=N), 1254 (C-N); Anal. Calcd for C_16_H_16_N_2_O_3_, C = 67.59, H = 5.37, N = 9.85, O = 16.88, Found C = 67.58, H = 5.68, N = 9.84, O = 16.89; EI MS *m/z* (% rel. abund.): 284.

*(E)-N'-(4-Hydroxy-3-methoxybenzylidene)-2,4-dimethylbenzohydrazide* (**8**). Yield: 0.494 g (83%); ^1^H-NMR (DMSO-*d_6_*): δ 11.45 (s, 1H, NH), 9.50 (s, 1H, OH), 8.19 (s, 1H, ArCH=N-R), 7.35 (d, 1H, *J*_6,5_ = 7.5 Hz, H-6), 7.31 (d, 1H, *J*_2',6'_ = 2.0 Hz, H-2'), 7.12 (s, 1H, H-3), 7.10 (d, 1H, *J*_5,6_ = 7.5 Hz, H-5), 7.07 (dd, 1H, *J*_6',2'_ = 2.0, *J*_6',5'_ = 8.0 Hz, H-6'), 6.84 (d 1H, *J*_5',6'_ = 8.0 Hz, H-5'), 2.36 (s, 3H, CH_3_), 2.32 (s, 3H, CH_3_), IR (KBr, cm^−1^): 3405 (OH), 3335 (N-H), 1659 (C=O), 1633 (C=N), 1249 (C-N); Anal. Calcd for C_17_H_18_N_2_O_3_, C = 68.45, H = 5.37, N = 9.39, O = 16.09, Found C = 68.46, H = 6.08, N = 9.40, O = 16.10; EI MS *m/z* (% rel. abund.): 298.

*(E)-N'-(4-Hydroxybenzylidene)-2,4-dimethylbenzohydrazide* (**9**). Yield: 0.493 g (92%); ^1^H-NMR (DMSO-*d_6_*): δ 11.43 (s, 1H, NH), 9.87 (s, 1H, OH), 8.20 (s, 1H, ArCH=N-R), 7.54 (d, 2H, *J*_2',3'_ = *J*_6',5'_ = 8.0 Hz, H-2',H-6'), 7.33 (d, 1H, *J*_6,5_ = 7.5 Hz, H-6), 7.17 (s, 1H, H-3), 7.11 (d, 1H, *J*_5,6_ = 7.5 Hz, H-5), 6.84 (d, 2H, *J*_3',2'_ = *J*_5',6'_ = 8.0 Hz, H-3',H-5'), 2.35 (s, 3H, CH_3_), 2.32 (s, 3H, CH_3_), IR (KBr, cm^−1^): 3410 (OH), 3325 (N-H), 1663 (C=O), 1636 (C=N), 1254 (C-N); Anal. Calcd for C_16_H_16_N_2_O_2_, C = 71.62, H = 6.01, N = 10.44, O = 11.93, Found C = 71.61, H = 6.02, N = 10.45, O = 11.94; EI MS *m/z* (% rel. abund.): 268.

*(E)-N'-(2-Hydroxybenzylidene)-2,4-dimethylbenzohydrazide* (**10**). Yield: 0.471g (88%); ^1^H-NMR (DMSO-*d_6_*): δ 11.11 (s, 1H, NH), 9.25 (s, 1H, OH), 9.01 (s, 1H, ArCH=N-R), 7.71 (dd, 1H, *J*_3',4'_ = 6.5, *J*_3',5'_ = 6.5 Hz, H-3') 7.43–7.39 (m, 2H, H-6',H-6), 7.17 (s, 1H, H-3), 7.11 (d, 1H, *J*_5,6_ = 7.5 Hz, H-5), 6.99–6.94 (m, 2H, H-4',H-5'), 2.38 (s, 3H, CH_3_), 2.33 (s, 3H, CH_3_); IR (KBr, cm^−1^): 3415 (OH), 3330 (N-H), 1663 (C=O), 1638 (C=N), 1258 (C-N); Anal. Calcd for C_16_H_16_N_2_O_2_, C = 71.62, H = 6.01, N = 10.44, O = 11.93, Found C = 71.60, H = 6.03, N = 10.44, O = 11.95; EI MS *m/z* (% rel. abund.): 268.

*(E)-N'-(3-Hydroxy-4-methoxybenzylidene)-2,4-dimethylbenzohydrazide* (**11**). Yield: 0.522 g (90%); ^1^H-NMR (DMSO-*d_6_*): *δ* 11.45 (s, 1H, NH), 9.26 (s, 1H, OH), 8.15 (s, 1H, ArCH=N-R), 7.34 (d, 1H, *J*_6,5_ = 7.5 Hz, H-6), 7.32 (d, 1H, *J*_2',6'_ = 2.0 Hz, H-2'), 7.17 (s, 1H, H-3), 7.10 (d, 1H, *J*_5,6_ = 7.5 Hz, H-5), 7.08 (dd, 1H, *J*_6',2'_ = 2.0, *J*_6',5'_ = 7.0, H-6'), 7.04 (d, 1H, *J*_5',6'_ = 7.0, H-5'), 2.35 (s, 3H, CH_3_), 2.32 (s, 3H, CH_3_), IR (KBr, cm^−1^): 3950 (OH), 3320 (N-H), 1658 (C=O), 1630 (C=N), 1254 (C-N); Anal. Calcd for C_17_H_18_N_2_O_3_, C = 68.45, H = 5.37, N = 9.39, O = 16.09, Found C = 68.46, H = 6.08, N = 9.40, O = 16.10; EI MS *m/z* (% rel. abund.): 298.

*(E)-N'-(3-Bromo-4-hydroxybenzylidene)-2,4-dimethylbenzohydrazide* (**12**). Yield: 0.602 g (87%); ^1^H-NMR (DMSO-*d_6_*): δ 11.35 (s, 1H, NH), 10.57 (s, 1H, OH), 8.30 (s, 1H, ArCH=N-R), 7.87 (d, 1H, *J*_2',6'_ = 2.0 Hz, H-2'), 7.58 (dd, 1H, *J*_6',2'_ = 2.0, *J*_6',5'_ = 7.0, H-6'), 7.32 (d, 1H, *J*_6,5_ = 7.5 Hz, H-6), 7.15 (s, 1H, H-3), 7.09 (d, 1H, *J*_5,6_ = 7.5 Hz, H-5), 7.05 (d, 1H, *J*_6',5'_ = 7.0, H-5'), 2.35 (s, 3H, CH_3_), 2.32 (s, 3H, CH_3_); IR (KBr, cm^−1^): 3954 (OH), 3318 (N-H), 1655 (C=O), 1635 (C=N), 1250 (C-N); Anal. Calcd for C_16_H_15_BrN_2_O_2_, C = 55.35, H = 4.35, N = 8.07, O = 9.22, Found C = 55.36, H = 4.35, N = 8.08, O = 9.21; EI MS *m/z* (% rel. abund.): 346.

*(E)-N'-(2-Hydroxy-5-methoxybenzylidene)-2,4-dimethylbenzohydrazide* (**13**). Yield: 0.536 g (90%); ^1^H-NMR (DMSO-*d_6_*): δ 11.88 (s, 1H, NH), 10.66 (s, 1H, OH), 8.48 (s, 1H, ArCH=N-R), 7.40 (d, 1H, *J*_6,5_ = 7.5 Hz, H-6), 7.14–7.09 (m, 1H, H-5, H-3, H-6'), 6.92 (d, 1H, *J*_3',4'_ = 7.0, H-3'), 6.87 (d, 1H, *J*_4',3'_ = 7.0, H-4'), 2.38 (s, 3H, CH_3_), 2.33 (s, 3H, CH_3_); IR (KBr, cm^−1^): 3350 (OH), 3310 (N-H), 1650 (C=O), 1632 (C=N), 1248 (C-N); Anal. Calcd for C_17_H_18_N_2_O_3_, C = 68.45, H = 5.37, N = 9.39, O = 16.09, Found C = 68.46, H = 6.08, N = 9.40, O = 16.10; EI MS *m/z* (% rel. abund.): 298.

*(E)-N'-(3-Hydroxy-2-iodo-4-methoxybenzylidene)-2,4-dimethylbenzohydrazide* (**14**). Yield: 0.738 g (87%); ^1^H-NMR (DMSO-*d_6_*): δ 11.88 (s, 1H, NH), 9.21 (s, 1H, OH), 8.48 (s, 1H, ArCH=N-R), 7.36 (d, 1H, *J*_6,5_ = 7.5 Hz, H-6), 7.16 (s, 1H, H-3), 7.10 (d, 1H, *J*_5,6_ = 7.5 Hz, H-5), 6.85 (d, 1H, *J*_6',5'_ = 7.0, H-6'), 6.68 (d, 1H, *J*_5',6'_ = 7.0, H-5'), 3.84 (s, 3H, OCH_3_), 2.35 (s, 3H, CH_3_), 2.32 (s, 3H, CH_3_); IR (KBr, cm^−1^): 3350 (OH), 3310 (N-H), 1650 (C=O), 1632 (C=N), 1248 (C-N); Anal. Calcd for C_17_H_17_IN_2_O_3_, C = 48.13, H = 4.04, N = 6.60, O = 11.31, Found C = 48.14, H = 4.05, N = 6.62, O = 11.33; EI MS *m/z* (% rel. abund.): 424.

*(E)-N'-(2-Hydroxy-4-methoxybenzylidene)-2,4-dimethylbenzohydrazide* (**15**). Yield: 0.488 g (82%); ^1^H-NMR (DMSO-*d_6_*): δ 11.78 (s, 1H, NH), 11.60 (s, 1H, OH), 8.41 (s, 1H, ArCH=N-R), 7.40 (d, 1H, *J*_6,5_ = 7.5 Hz, H-6), 7.38 (d, 1H, *J*_3',5'_ = 2.0 Hz, H-3'), 7.14 (s, 1H, H-3), 7.11 (d, 1H, *J*_5,6_ = 7.5 Hz, H-5), 6.53 (d, 1H, *J*_5',6'_ = 7.5 Hz, H-5'), 6.50 (d, 1H, *J*_6',5'_ = 7.5 Hz, H-6'), 2.37 (s, 3H, CH_3_), 2.33 (s, 3H, CH_3_); IR (KBr, cm^−1^): 3390 (OH), 3300 (N-H), 1655 (C=O), 1630 (C=N), 1250 (C-N); Anal. Calcd for C_17_H_18_N_2_O_3_, C = 68.45, H = 5.37, N = 9.39, O = 16.09, Found C = 68.46, H = 6.08, N = 9.40, O = 16.10; EI MS *m/z* (% rel. abund.): 298.

*(E)-N'-(3,5-Dimethoxybenzylidene)-2,4-dimethylbenzohydrazide* (**16**). Yield: 0.511 g (82%); ^1^H-NMR (DMSO-*d_6_*): δ 11.67 (s, 1H, NH), 8.23 (s, 1H, ArCH=N-R), 7.36 (d, 1H, *J*_6,5_ = 7.5 Hz, H-6), 7.14 (s, 1H, H-3), 7.11 (d, 1H, *J*_5,6_ = 7.5 Hz, H-5), 6.86 (s, 2H, H-2', H-6'), 6.57 (s, 1H, H-4'), 3.86 (s, 6H, 2× OCH_3_), 2.36 (s, 3H, CH_3_), 2.33 (s, 3H, CH_3_); IR (KBr, cm^−1^): 3310 (N-H), 1650 (C=O), 1628 (C=N), 1252 (C-N); Anal. Calcd for C_18_H_20_N_2_O_3_, C = 69.21, H = 6.45, N = 8.97, O = 15.37, Found C = 69.20, H = 6.44, N = 8.98, O = 15.38; EI MS *m/z* (% rel. abund.): 312.

*(E)-N'-(3,4-Dimethoxybenzylidene)-2,4-dimethylbenzohydrazide* (**17**). Yield: 0.524 g (84%); ^1^H-NMR (DMSO-*d_6_*): δ 11.40 (s, 1H, NH), 8.20 (s, 1H, ArCH=N-R), 7.35 (d, 1H, *J*_6,5_ = 7.5 Hz, H-6), 7.18 (s, 1H, H-2′) 7.12 (s, 1H, H-3), 7.10 (d, 1H, *J*_5,6_ = 7.5 Hz, H-5), 6.11 (d, 1H, *J*_5',6'_ = 8.0 Hz, H-5'), 6.81 (d, 1H, *J*_6',5'_ = 8.0 Hz, H-6'), 3.90 (s, 3H, OCH3), 3.85 (s, 3H, OCH3), 2.38 (s, 3H, CH_3_), 2.34 (s, 3H, CH_3_); IR (KBr, cm^−1^): 3340 (N-H), 1653 (C=O), 1634 (C=N), 1256 (C-N); Anal. Calcd for C_18_H_20_N_2_O_3_, C = 69.21, H = 6.45, N = 8.97, O = 15.37, Found C = 69.20, H = 6.44, N = 8.98, O = 15.38; EI MS *m/z* (% rel. abund.): 312.

*(E)-N'-(3-Methoxybenzylidene)-2,4-dimethylbenzohydrazide* (**18**). Yield: 0.468 g (83%); ^1^H-NMR (DMSO-*d_6_*): δ 11.66 (s, 1H, NH), 8.28 (s, 1H, ArCH=N-R), 7.45-7.38 (m, 4H, H-4', H-5', H-6', H-6), 7.13 (s, 1H, H-3), 7.11 (d, 1H, *J*_5,6_ = 7.5 Hz, H-5), 7.08 (d, 1H, *J*_2',6'_ = 2.0 Hz, H-2'), 3.88 (s, 3H, OCH3), 2.36 (s, 3H, CH_3_), 2.33 (s, 3H, CH_3_); IR (KBr, cm^−1^): 3324 (N-H), 1658 (C=O), 1638 (C=N), 1251 (C-N); Anal. Calcd for C_17_H_18_N_2_O_2_, C = 72.32, H = 6.43, N = 9.92, O = 11.33, Found C = 72.33, H = 6.44, N = 9.91, O = 11.34; EI MS *m/z* (% rel. abund.): 282.

*(E)-N'-(2-Methylbenzylidene)-2,4-dimethylbenzohydrazide* (**19**) [40]; Yield: 0.452 g (85%); ^1^H-NMR (DMSO-*d_6_*): δ 11.66 (s, 1H, NH), 8.28 (s, 1H, ArCH=N-R), 7.35-7.32 (m, 4H, H-3', H-4', H-5', H-6), 7.12 (s, 1H, H-3), 7.09 (d, 1H, *J*_5,6_ = 7.5 Hz, H-5), 7.01 (d, 1H, *J*_6',5'_ = 8.0 Hz, H-6'), 2.38 (s, 3H, CH3), 2.35 (s, 3H, CH_3_), 2.32 (s, 3H, CH_3_); IR (KBr, cm^−1^): 3330 (N-H), 1652 (C=O), 1642 (C=N), 1255 (C-N); Anal. Calcd for C_17_H_18_N_2_O, C = 76.66, H = 6.81, N = 10.52, O = 6.01, Found C = 76.68, H = 6.82, N = 10.50, O = 6.02; EI MS *m/z* (% rel. abund.): 266.

*(E)-N'-(4-Bromo-3-florobenzylidene)-2,4-dimethylbenzohydrazide* (**20**). Yield: 0.605 g (87%); ^1^H-NMR (DMSO-*d_6_*): δ 11.85 (s, 1H, NH), 8.82 (s, 1H, ArCH=N-R), 7.45–7.38 (m, 3H, H-5', H-6', H-6), 7.21 (d, 1H, *J*_2',6'_ = 2.0 Hz, H-2'), 7.11 (s, 1H, H-3), 7.08 (d, 1H, *J*_5,6_ = 7.5 Hz, H-5), 2.38 (s, 3H, CH_3_), 2.34 (s, 3H, CH_3_); IR (KBr, cm^−1^): 3310 (N-H), 1651 (C=O), 1632 (C=N), 1247 (C-N); Anal. Calcd for C_16_H_14_BrFN_2_O, C = 55.03, H = 4.04, N = 8.02, O = 4.59, Found C = 55.03, H = 4.03, N = 8.04, O = 4.59; EI MS *m/z* (% rel. abund.): 348.

*(E)-N'-(4-Methoxybenzylidene)-2,4-dimethylbenzohydrazide* (**21**). Yield: 0.496 g (88%); ^1^H-NMR (DMSO-*d_6_*): δ 11.30 (s, 1H, NH), 8.10 (s, 1H, ArCH=N-R), 7.62 (d, 2H, *J*_2',3'_ = *J*_6',5'_ = 8.0 Hz, H-2', H-6'), 7.34 (d, 1H, *J*_6,5_ = 7.0 Hz, H-6), 7.11 (s, 1H, H-3), 7.08 (d, 1H, *J*_5,6_ = 7.0 Hz, H-5), 6.92 (d, 2H, *J*_3',2'_ = *J*_5',6'_ = 8.0 Hz, H-3', H-5'), 3.84 (s, 3H, OCH3), 2.36 (s, 3H, CH_3_), 2.33 (s, 3H, CH_3_); IR (KBr, cm^−1^): 3342 (N-H), 1661 (C=O), 1640 (C=N), 1254 (C-N); Anal. Calcd for C_17_H_18_N_2_O_2_, C = 72.32, H = 6.43, N = 9.92, O = 11.33, Found C = 72.33, H = 6.45, N = 9.92, O = 11.32; EI MS *m/z* (% rel. abund.): 282.

*(E)-2,4-Dimethyl-N'-((pyridin-3-yl)methylene)benzohydrazide* (**22**). Yield: 0.455 g (90%); ^1^H-NMR (DMSO-*d_6_*): δ 11.82 (s, 1H, NH), 8.84 (s, 1H, ArCH=N-R), 8.61 (s, 1H, H-2'), 8.31 (s, 1H, H-6'), 8.13 (d, 1H, *J*_4',5'_ = 7.0 Hz, H-4'), 7.49 (d, 1H, *J*_5',4'_ = 7.0 Hz, H-5'), 7.38 (d, 1H, *J*_6,5_ = 7.0 Hz, H-6), 7.14 (s, 1H, H-3), 7.12 (d, 1H, *J*_5,6_ = 7.5 Hz, H-5), 2.37 (s, 3H, CH_3_), 2.33 (s, 3H, CH_3_); IR (KBr, cm^−1^): 3360 (N-H), 1670 (C=O), 1645 (C=N), 1258 (C-N); Anal. Calcd for C_15_H_15_N_3_O, C = 71.13, H = 5.97, N = 16.59, O = 6.32, Found C = 71.14, H = 5.98, N = 16.61, O = 6.33; EI MS *m/z* (% rel. abund.): 253.

*(E)-N'-(4-Methylbenzylidene)-2,4-dimethylbenzohydrazide* (**23**). Yield: 0.490 g (92%); ^1^H-NMR (DMSO-*d_6_*): δ 11.58 (s, 1H, NH), 8.27 (s, 1H, ArCH=N-R), 7.61 (d, 2H, *J*_2',3'_ = *J*_6',5'_ = 7.5 Hz, H-2', H-6'), 7.35 (d, 1H, *J*_6,5_ = 7.5 Hz, H-6), 7.32 (d, 2H, *J*_3',2'_ = *J*_5',6'_ = 7.5 Hz, H-3', H-5'), 7.17 (s, 1H, H-3), 7.13 (d, 1H, *J*_5,6_ = 7.5 Hz, H-5), 2.37 (s, 3H, CH_3_), 2.33 (s, 6H, 2× CH_3_), IR (KBr, cm^−1^): 3345 (N-H), 1658 (C=O), 1632 (C=N), 1250 (C-N); Anal. Calcd for C_17_H_18_N_2_O, C = 76.66, H = 6.81, N = 10.52, O = 6.01, Found C = 76.67, H = 6.82, N = 10.50, O = 6.02; EI MS *m/z* (% rel. abund.): 266.

*(E)-2,4-Dimethyl-N'-((pyridin-4-yl)methylene)benzohydrazide* (**24**). Yield: 0.455 g (90%); ^1^H-NMR (DMSO-*d_6_*): δ 11.99 (s, 1H, NH), 8.65 (d, 2H, *J*_2',3'_ = *J*_6',5'_ = 6.0 Hz, H-2', H-6'), 8.30 (s, 1H, ArCH=N-R), 7.66 (d, 2H, *J*_3',2'_ = *J*_5',6'_ = 6.0 Hz, H-3', H-5'), 7.40 (d, 1H, *J*_6,5_ = 7.5 Hz, H-6), 7.15 (s, 1H, H-3), 7.12 (d, 1H, *J*_5,6_ = 7.5 Hz, H-5), 2.38 (s, 3H, CH_3_), 2.32 (s, 3H, CH_3_), IR (KBr, cm^−1^): 3350 (N-H), 1654 (C=O), 1638 (C=N), 1254 (C-N); Anal. Calcd for C_15_H_15_N_3_O, C = 71.13, H = 5.97, N = 16.59, O = 6.32, Found C = 71.13, H = 6.00, N = 16.62, O = 6.32; EI MS *m/z* (% rel. abund.): 253.

*(E)-2,4-Dimethyl-N'-((pyridin-2-yl)methylene)benzohydrazide* (**25**). Yield: 0.414 g (82%); ^1^H-NMR (DMSO-*d_6_*): δ 11.87 (s, 1H, NH), 8.55 (d, 2H, *J*_6',5'_ = 6.0 Hz, H-6'), 8.25 (s, 1H, ArCH=N-R), 7.90–7.86 (m, 2H, H-4', H-5'), 7.38 (d, 1H, *J*_6,5_ = 7.0 Hz, H-6), 7.13 (s, 1H, H-3), 7.10 (d, 1H, *J*_5,6_ = 7.0 Hz, H-5), 6.95 (d, 2H, *J*_3',4'_ = 6.5 Hz, H-3'), 2.37 (s, 3H, CH_3_), 2.33 (s, 3H, CH_3_), IR (KBr, cm^−1^): 3342 (N-H), 1656 (C=O), 1636 (C=N), 1253 (C-N); Anal. Calcd for C_15_H_15_N_3_O, C = 71.13, H = 5.97, N = 16.59, O = 6.32, Found C = 71.15, H = 6.00, N = 16.62, O = 6.32; EI MS *m/z* (% rel. abund.): 253.

*(E)-2,4-Dimethyl-N'-((thiophen-2-yl)methylene)benzohydrazide* (**26**). Yield: 0.454 g (88%); ^1^H-NMR (DMSO-*d_6_*): δ 11.63 (s, 1H, NH), 8.51 (s, 1H, ArCH=N-R), 7.66 (s, 1H, H-5'), 7.43 (s, 1H, H-2'), 7.35 (d, 1H, *J*_6,5_ = 7.5 Hz, H-6), 7.11–7.08 (m, 3H, H-3, H-5, H-4'), 2.36 (s, 3H, CH_3_), 2.33 (s, 3H, CH_3_), IR (KBr, cm^−1^): 3350 (N-H), 1654 (C=O), 1638 (C=N), 1254 (C-N); Anal. Calcd for C_14_H_14_N_2_OS, C = 65.09, H = 5.46, N = 10.84, O = 6.19, Found C = 65.08, H = 5.48, N = 10.83, O = 6.18; EI MS *m/z* (% rel. abund.): 258.

*Methyl 4-((E)-(2,4-dimethylbenzoylimino)methyl)benzoate* (**27**). Yield: 0.558 g (90%); ^1^H-NMR (DMSO-*d_6_*): δ 11.83 (s, 1H, NH), 8.36 (s, 1H, ArCH=N-R), 8.04 (d, 2H, *J*_2',3'_ = *J*_6',5'_ = 7.5 Hz, H-2', H-6'), 7.86 (d, 2H, *J*_3',2'_ = *J*_5',6'_ = 7.5 Hz, H-3', H-5'), 7.38 (d, 1H, *J*_6,5_ = 7.5 Hz, H-6), 7.14 (s, 1H, H-3), 7.11 (d, 1H, *J*_5,6_ = 7.5 Hz, H-5), 3.88 (s, 3H, OCH_3_), 2.36 (s, 3H, CH_3_), 2.33 (s, 3H, CH_3_), IR (KBr, cm^−1^): 3345 (N-H), 1658 (C=O), 1632 (C=N), 1250 (C-N); Anal. Calcd for C_18_H_18_N_2_O_3_, C = 69.66, H = 5.85, N = 9.03, O = 15.47, Found C = 69.67, H = 5.86, N = 9.04, O = 15.48; EI MS *m/z* (% rel. abund.): 310.

*(E)-N'-(4-Chlorobenzylidene)-2,4-dimethylbenzohydrazide* (**28**). Yield: 0.526 g (92%); ^1^H-NMR (DMSO-*d_6_*): δ 11.72 (s, 1H, NH), 8.30 (s, 1H, ArCH=N-R), 7.74 (d, 2H, *J*_2',3'_ = *J*_6',5'_ = 7.5 Hz, H-2', H-6'), 7.54 (d, 2H, *J*_3',2'_ = *J*_5',6'_ = 7.5 Hz, H-3', H-5'), 7.37 (d, 1H, *J*_6,5_ = 7.5 Hz, H-6), 7.13 (s, 1H, H-3), 7.10 (d, 1H, *J*_5,6_ = 7.5 Hz, H-5), 2.36 (s, 3H, CH_3_), 2.33 (s, 3H, CH_3_), IR (KBr, cm^−1^): 3345 (N-H), 1658 (C=O), 1632 (C=N), 1250 (C-N); Anal. Calcd for C_16_H_15_ClN_2_O, C = 67.02, H = 5.27, N = 9.77, O = 5.58, Found C = 67.03, H = 5.28, N = 9.78, O = 5.57; EI MS *m/z* (% rel. abund.): 286.

*Methyl 2-((E)-(2,4-dimethylbenzoylimino)methyl)-3,5-dimethoxybenzoate* (**29**). Yield: 0.673 g (91%); ^1^H-NMR (DMSO-*d_6_*): δ 11.62 (s, 1H, NH), 8.41 (s, 1H, ArCH=N-R), 7.36 (d, 1H, *J*_6,5_ = 7.0 Hz, H-6), 7.11–7.08 (m, 3H, H-3, H-5, H-6'), 6.72 (d, 1H, *J*_4',6'_ = 2.0 Hz, H-4'), 3.89 (s, 3H, R-COOCH_3_), 3.81(s, 6H, 2× OCH_3_), 2.37 (s, 3H, CH_3_), 2.34 (s, 3H, CH_3_), IR (KBr, cm^−1^): 3345 (N-H), 1658 (C=O), 1632 (C=N), 1250 (C-N); Anal. Calcd for C_20_H_22_N_2_O_5_, C = 64.85, H = 5.99, N = 7.56, O = 21.60, Found C = 64.87, H = 5.97, N = 7.58, O = 21.62; EI MS *m/z* (% rel. abund.): 370.

*(E)-N'-(4-Nitrobenzylidene)-2,4-dimethylbenzohydrazide* (**30**). Yield: 0.499 g (84%); ^1^H-NMR (DMSO-*d_6_*): δ 11.97 (s, 1H, NH), 8.87 (s, 1H, ArCH=N-R), 8.31 (d, 2H, *J*_2',3'_ = *J*_6',5'_ = 8.5 Hz, H-2', H-6'), 7.99 (d, 2H, *J*_3',2'_ = *J*_5',6'_ = 8.5 Hz, H-3', H-5'), 7.40 (d, 1H, *J*_6,5_ = 7.5 Hz, H-6), 7.15 (s, 1H, H-3), 7.12 (d, 1H, *J*_5,6_ = 7.5 Hz, H-5), 2.38 (s, 3H, CH_3_), 2.34 (s, 3H, CH_3_), IR (KBr, cm^−1^): 3345 (N-H), 1658 (C=O), 1632 (C=N), 1250 (C-N); Anal. Calcd for C_16_H_15_N_3_O_3_, C = 64.64, H = 5.09, N = 14.13, O = 16.14, Found C = 64.65, H = 5.09, N = 14.14, O = 16.15; EI MS *m/z* (% rel. abund.): 297.

## 5. Conclusions

In this study we synthesized thirty (30) 2,4-dimethylbenzoylhydrazones derivatives. Out of thirty compounds, six (6) compounds showed better activities than the reference compound (*n*-propyl gallate) for DPPH activity and three compounds showed better activity than standard toward superoxide anion. Along with these results we also reported three new crystal structures. We believe that these molecules merit further study of their antiaging, antioxidant as well as potential anticancer activities.
